# Supplementary material for: Impaired α-Synuclein aggregate clearance in neuronal cells drive their spread to microglia through tunneling nanotubes
Source: Nat Commun. 2026 Mar 12;17:3832. doi: 10.1038/s41467-026-69930-y (PMC13121836; doi:10.1038/s41467-026-69930-y)
Supplement: Supplementary file 1 — Supplementary information [file 41467_2026_69930_MOESM1_ESM.pdf]

Supplementary Information – Supplementary figures and legends

Impaired α-Synuclein aggregate clearance in neuronal cells drive their spread to microglia through tunneling nanotubes

Authors

Ranabir Chakraborty[1,2], Francesca Palese[1], Philippa Samella[1,3], Veronica Testa[4,#], Jara Montero-Muñoz[4,#], Sylvie Syan[1], Takashi Nonaka[5], Masato Hasegawa[5], Antonella Consiglio[4], Chiara Zurzolo[1,6,*]

Affiliations

[1]Institut Pasteur, Université Paris Cité, CNRS UMR 3691, Membrane Traffic and Pathogenesis, F-75015 Paris, France
[2]Université Paris-Saclay, 91190 Gif-sur-Yvette, France
[3]Trinity College, University of Cambridge, Cambridge, CB2 1TQ, United Kingdom
[4]Department of Pathology and Experimental Therapeutics, Bellvitge University Hospital-IDIBELL, 08908 Hospitalet de Llobregat, Spain; Institute of Biomedicine of the University of Barcelona (IBUB), Carrer Baldiri Reixac 15-21, Barcelona 08028, Spain
[5]Dementia Research Project, Tokyo Metropolitan Institute of Medical Science, Tokyo, Japan
[6]Department of Molecular Medicine and Medical Biotechnology, University of Naples Federico II, Naples, Italy
[#]Equal contributions
[*]Correspondence: chiara.zurzolo@pasteur.fr

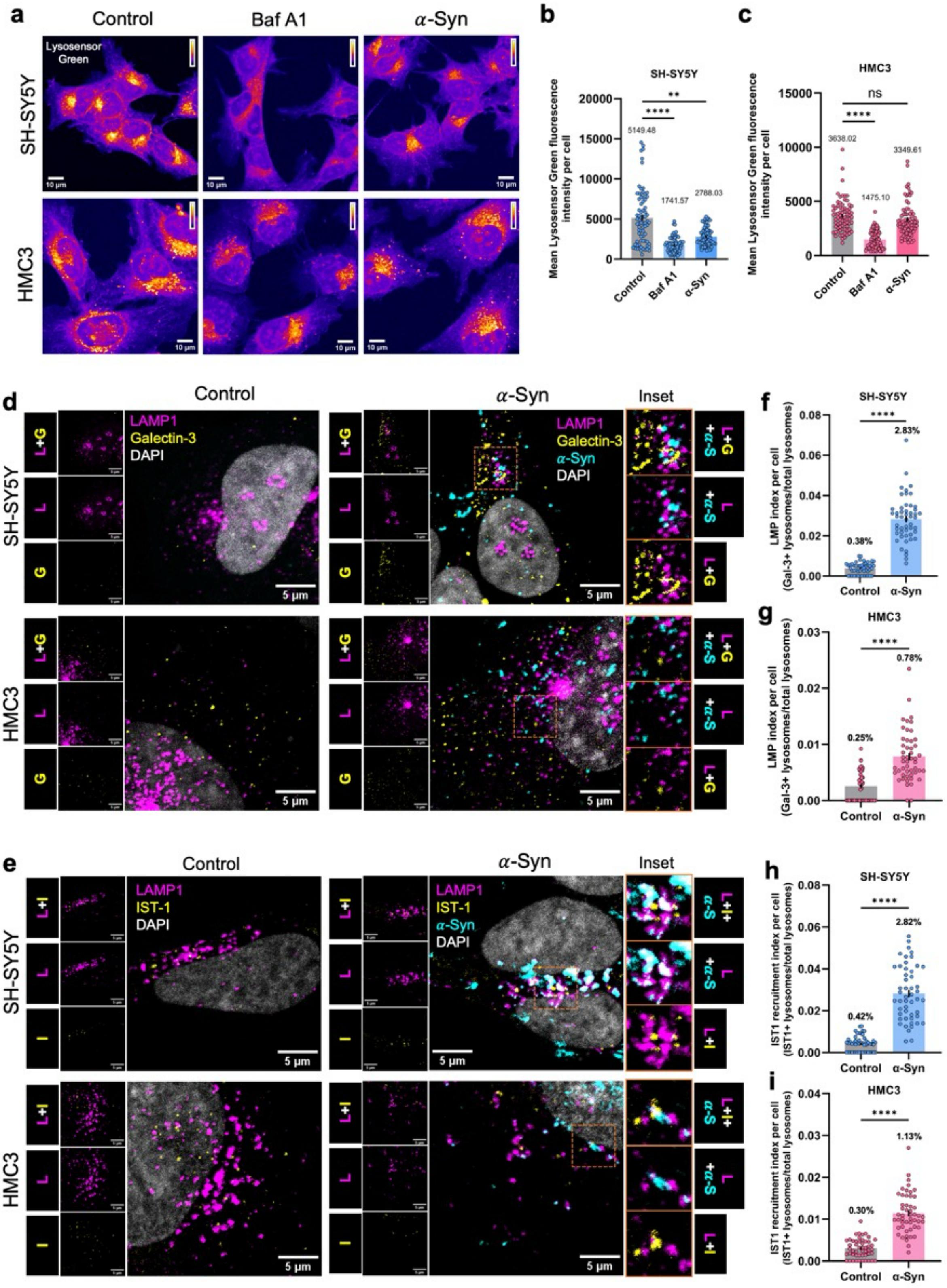

a
Control
Baf A1
α-Syn
Lysosensor Green
SH-SY5Y
HMC3
10 μm
b
SH-SY5Y
Mean Lysosensor Green fluorescence intensity per cell
5149.48
1741.57
2788.03
****
**
Control
Baf A1
α-Syn
c
HMC3
3638.02
1475.10
3349.61
****
ns
d
Control
α-Syn
Inset
LAMP1
Galectin-3
α-Syn
DAPI
L+G
L
G
5 μm
f
SH-SY5Y
LMP index per cell (Gal-3+ lysosomes/total lysosomes)
0.38%
2.83%
****
g
HMC3
0.25%
0.78%
e
IST-1
L+I
I
h
IST1 recruitment index per cell (IST1+ lysosomes/total lysosomes)
0.42%
2.82%
i
0.30%
1.13%

Supplementary Figure 1. Lysosomal damage in neuronal and microglial cells. (**a**) Representative images of SH-SY5Y cells (upper panels) and HMC3 (lower panels) loaded with the pH-sensitive dye lysosensor green (fire LUT). (**b** and **c**) Quantification of mean lysosensor green fluorescence intensity in neuronal cells (**b**) and microglial cells (**c**). Error bars represent SEM. N=3, n=75 cells per group. Statistical significance was analyzed using Kruskal-Wallis test with Dunn's multiple comparison. For (**b**): **p=0.0015, ****$p<1x10^{-15}$. For (**c**): ns: p=0.3416; ****$p<1x10^{-15}$. (**d**) Representative images of SH-SY5Y cells (upper panels) and HMC3 cells (bottom panels) immunostained for Galectin-3 (yellow) and LAMP1 (magenta) in control and α-Syn (cyan) treated conditions. Insets show regions of triple colocalization of α-Syn-associated lysosome with its membrane permeabilized. (**e**) Representative images of SH-SY5Y cells (upper panels) and HMC3 cells (bottom panels) immunostained for IST-1 (yellow) and LAMP1 (magenta) in control and α-Syn (cyan) treated conditions. Insets show regions of triple colocalization of α-Syn-associated lysosome with recruited ESCRT-III related protein IST1 for potential membrane repair. (**f** and **g**) Quantification of the percentage of lysosomes per cell with membrane permeabilized in neuronal cells (**f**) and microglia (**g**). Error bars represent SEM. N=3, n=50 cells per group. Statistical significance was analyzed using two-sided Mann-Whitney U test. For (**f**), ****$p<1x10^{-15}$, and for (g), ****$p=7.16x10^{-11}$. (**h** and **i**) Quantification of the percentage of lysosomes per cell with IST1 recruitment in neuronal cells (**h**) and microglia (**i**). Error bars represent SEM. N=3, n=50 cells per group. Statistical significance was analyzed using two-sided Mann-Whitney U test. For (**h**) and (**i**), ****$p<1x10^{-15}$.

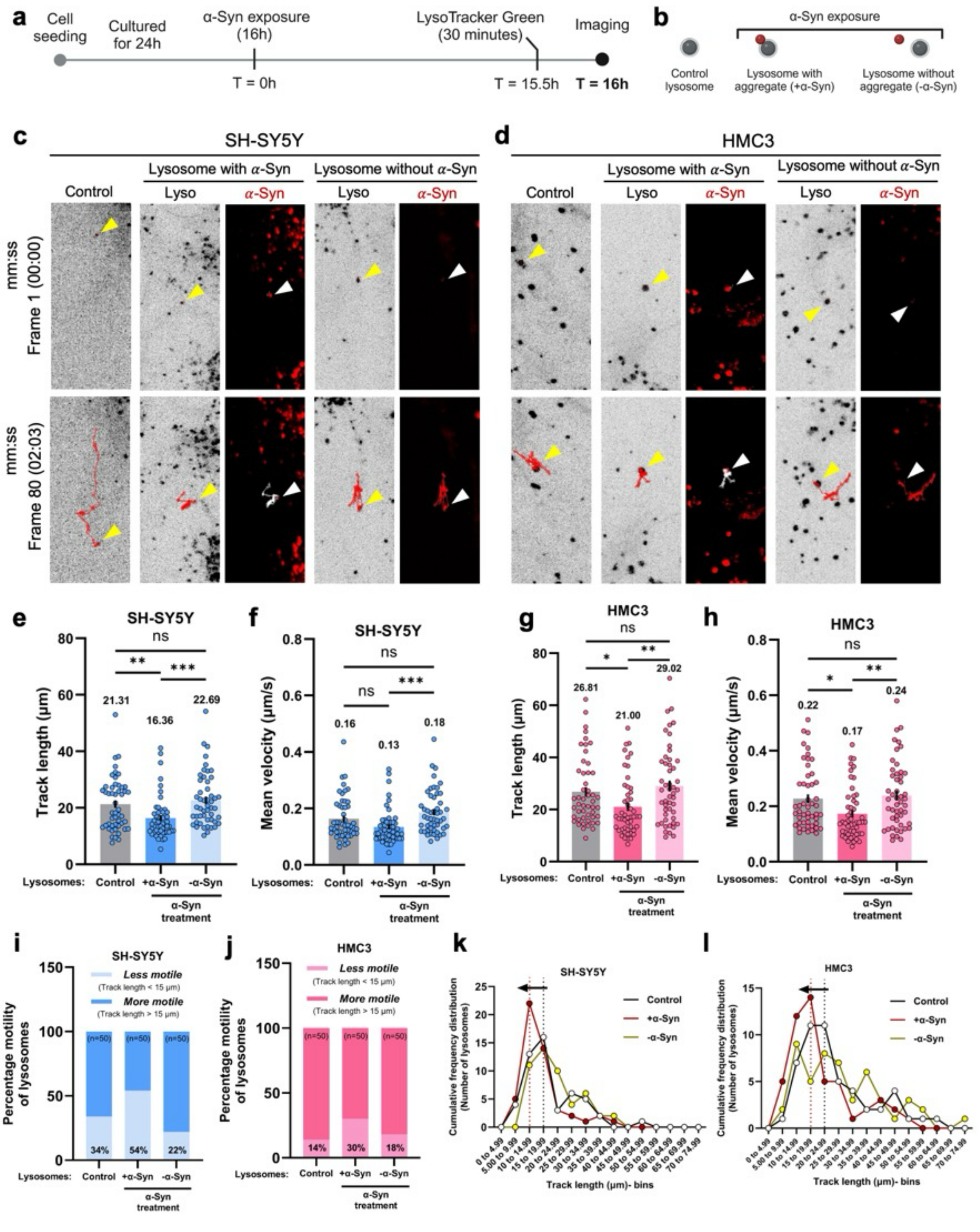
a
Cell seeding
Cultured for 24h
α-Syn exposure (16h)
T = 0h
LysoTracker Green (30 minutes)
T = 15.5h
Imaging
T = 16h
b
α-Syn exposure
Control lysosome
Lysosome with aggregate (+α-Syn)
Lysosome without aggregate (-α-Syn)
c
SH-SY5Y
Control
Lysosome with α-Syn
Lysosome without α-Syn
Lyso
α-Syn
Frame 1 (00:00) mm:ss
Frame 80 (02:03) mm:ss
d
HMC3
e
SH-SY5Y
Track length (μm)
21.31
16.36
22.69
ns
**
***
Lysosomes: Control +α-Syn -α-Syn
α-Syn treatment
f
SH-SY5Y
Mean velocity (μm/s)
0.16
0.13
0.18
ns
ns
***
g
HMC3
Track length (μm)
26.81
21.00
29.02
ns
*
**
h
HMC3
Mean velocity (μm/s)
0.22
0.17
0.24
ns
*
**
i
SH-SY5Y
Less motile (Track length < 15 μm)
More motile (Track length > 15 μm)
Percentage motility of lysosomes
(n=50)
34%
54%
22%
j
HMC3
Less motile (Track length < 15 μm)
More motile (Track length > 15 μm)
Percentage motility of lysosomes
(n=50)
14%
30%
18%
k
SH-SY5Y
Cumulative frequency distribution (Number of lysosomes)
Control
+α-Syn
-α-Syn
Track length (μm)- bins
l
HMC3
Cumulative frequency distribution (Number of lysosomes)
Control
+α-Syn
-α-Syn
Track length (μm)- bins

Supplementary Figure 2. Motility of lysosomes. (**a** and **b**) Schematic representation of the experimental (**a**), and analysis strategy (**b**) to track lysosome motility. Created in BioRender. Palese, F. (2026) https://BioRender.com/3zimbkm (**c** and **d**) Representative images of the first frame (upper panels) and last (80th) frame (lower panels) of tracked lysosomes for SH-SY5Y cells (**c**) and HMC3 cells (**d**). Arrowheads point towards the tracked lysosome. (**e**) Mean track lengths of lysosomes in neuronal cells. Statistical significance was analyzed using Kruskal-Wallis test with Dunn's multiple comparison. ns: non-significant ($p>0.9999$), **$p=0.0067$, ***$p=0.0002$. (**f**) Mean velocities of lysosomes in neuronal cells. Statistical significance was analyzed using Kruskal-Wallis test with Dunn's multiple comparison. ns: non-significant ($p=0.0664$ between control and +α-Syn groups, $p=0.2038$ for control and -α-Syn groups), ***$p=0.0001$. (**g**) Mean track lengths of lysosomes in microglia. Statistical significance was analyzed using Kruskal-Wallis test with Dunn's multiple comparison. ns: non-significant ($p>0.9999$), *$p=0.0319$, **$p=0.0052$. (**h**) Mean velocities of lysosomes in microglia. Statistical significance was analyzed using Kruskal-Wallis test with Dunn's multiple comparison. ns: non-significant ($p>0.9999$), *$p=0.0182$, **$p=0.0052$. (**i** and **j**) Proportion of lysosomes that travel less than 15 μm (less motile), or more than 15 μm (more motile) in different conditions for neuronal cells (**i**) and microglia (**j**). For quantifications in (**e-j**): N=3 independent experiments, n=50 individual lysosomes tracked per group. Mean values are mentioned within the graphs. Error bars represent SEM. (**k** and **l**) Cumulative frequency distribution of the 50 tracked lysosomes of SH-SY5Y (**k**) and HMC3 (**l**) cells that travel different distances, binned in clusters of 4.99 μm. Black vertical line indicates the peak point for control lysosomes, and red vertical line indicates the same for lysosomes associated with α-Syn. Black arrows within the graphs indicate a shift in the peak (less distance travelled by aggregate-associated lysosomes).

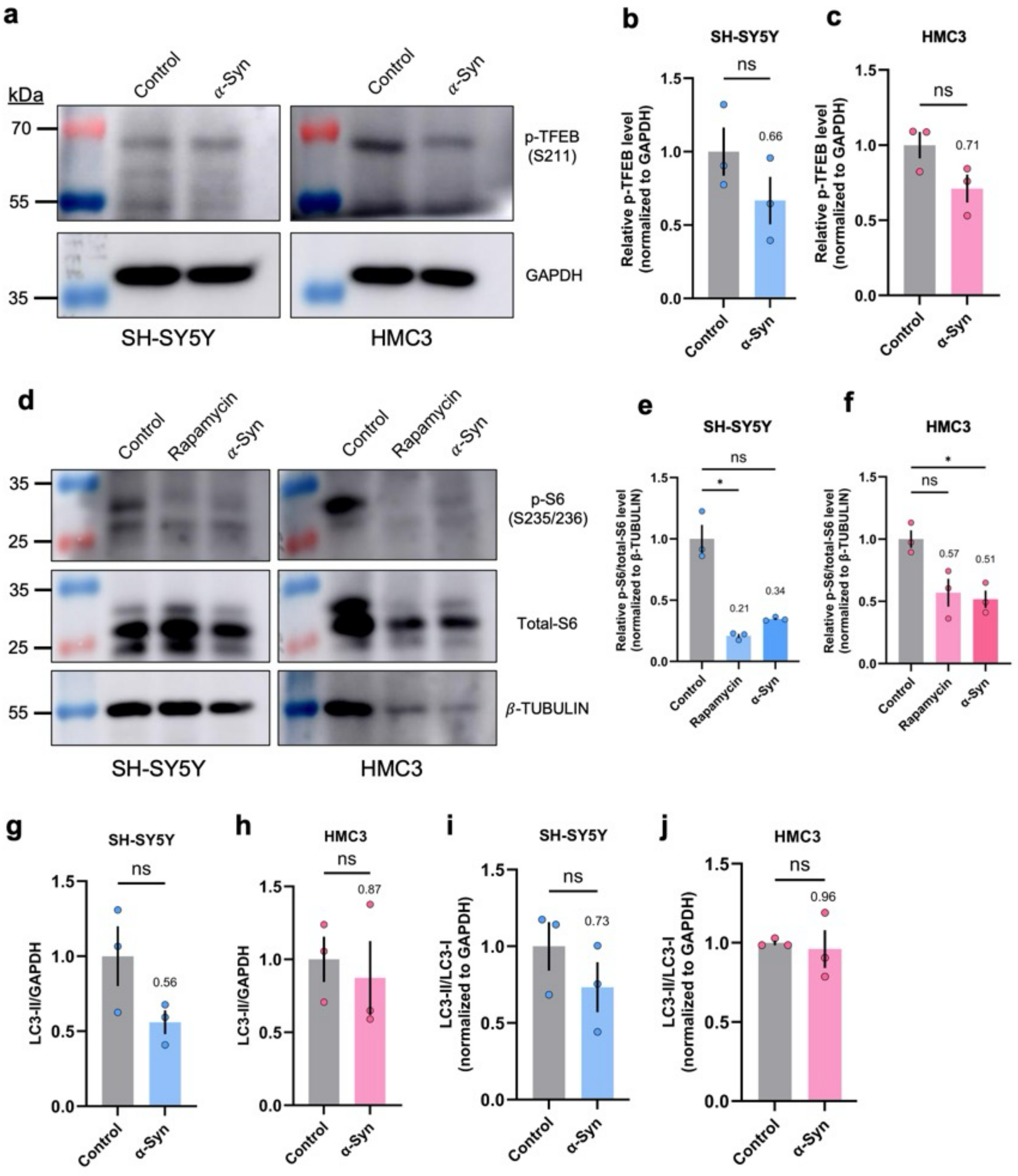

Supplementary Figure 3. Regulation of TFEB activity and mTOR activation upon α-Syn exposure. (**a**) Representative immunoblots of phosphorylation of TFEB in Ser211 residue. (**b** and **c**) Quantification of the relative phospho-S211-TFEB level normalized to GAPDH in neuronal cells (**b**) and microglia (**c**). Fold differences are mentioned within the graphs.

N=3 independent experiments. Error bars represent SEM. Statistical significance was analyzed using two-sided unpaired Student's t-test with Welch's correction. For (**b**): ns – p=0.2226; for (**c**): ns – p=0.0884. (d) Representative immunoblots of phosphorylation of S6 ribosomal protein in Ser235/236 residues, and total level of S6. (**e** and **f**) Quantification of the relative phospho-S235/236 levels normalized to total S6 level and β-TUBULIN in neuronal cells (**e**) and microglia (**f**). Fold differences are mentioned within the graphs. N=3 independent experiments. Error bars represent SEM. Statistical significance was analyzed using Brown-Forsythe One-Way ANOVA with Dunnett's T3 multiple comparisons. For (**e**): control versus rapamycin: *p=0.0430; control versus α-Syn: ns – p=0.0611. For (**f**): control versus rapamycin: ns – p=0.1076; control versus α-Syn: *p=0.0207. (**g** and **h**) Quantification of LC3B-II levels in high exposure blots from Fig. 4A in non-baf A1 treated conditions in neuronal cells (**g**) and microglia (**h**). Statistical significance was analyzed using two-sided unpaired Student's t-test. For (**g**): ns – p=0.1095; for (**h**) ns – p=0.6904. (**i** and **j**) Quantification of LC3B lipidation efficiency in non-bafA1 treated groups from high exposure blots in Fig. 4A in neuronal cells (**i**) and microglia (**j**). Statistical significance was analyzed using two-sided unpaired Student's t-test. For (**i**): ns – p=0.3058; for (**j**) ns – p=0.7644.

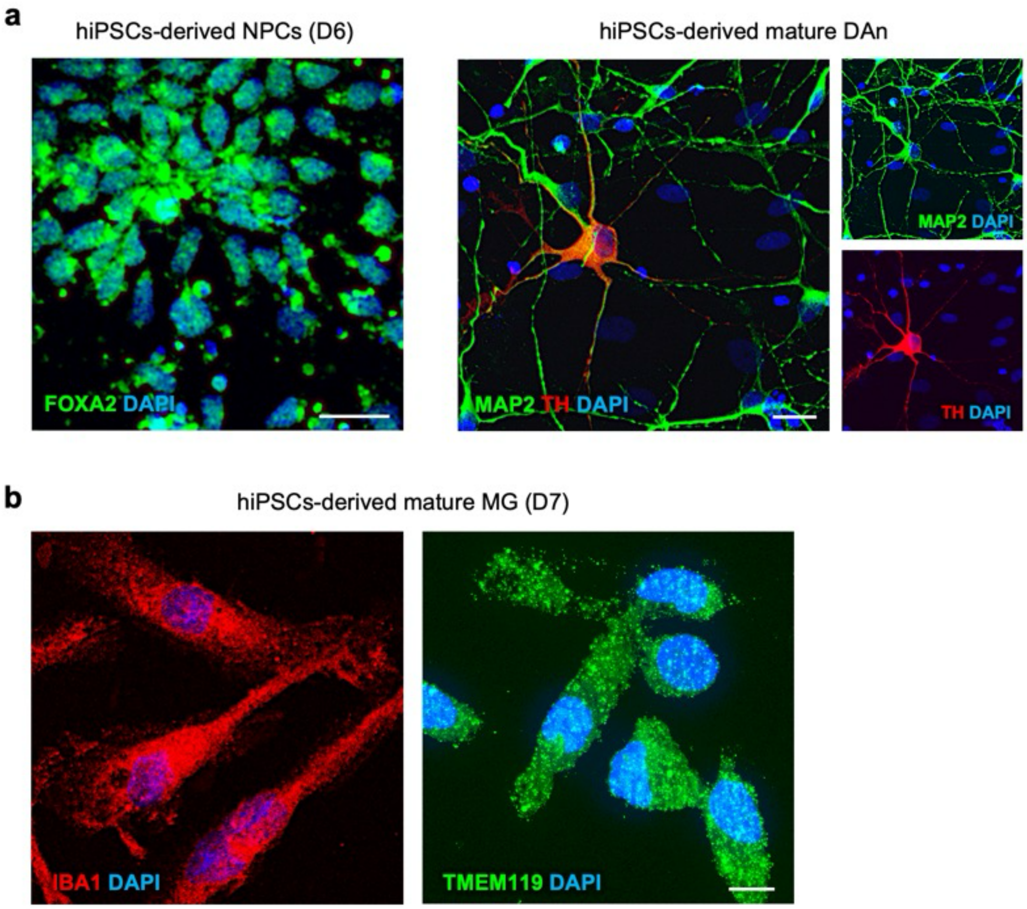

Supplementary figure 4. Characterization of hiPSC-derived cells. (**a**) Characterization of hiPSC-derived neural progenitor cells (NPCs; positive for FOXA2), and dopaminergic neurons (positive for tyrosine hydroxylase [TH], with MAP2 as a mature neuron marker). (**b**) Characterization of hiPSC-derived microglia (positive for canonical markers IBA1 and TMEM119).

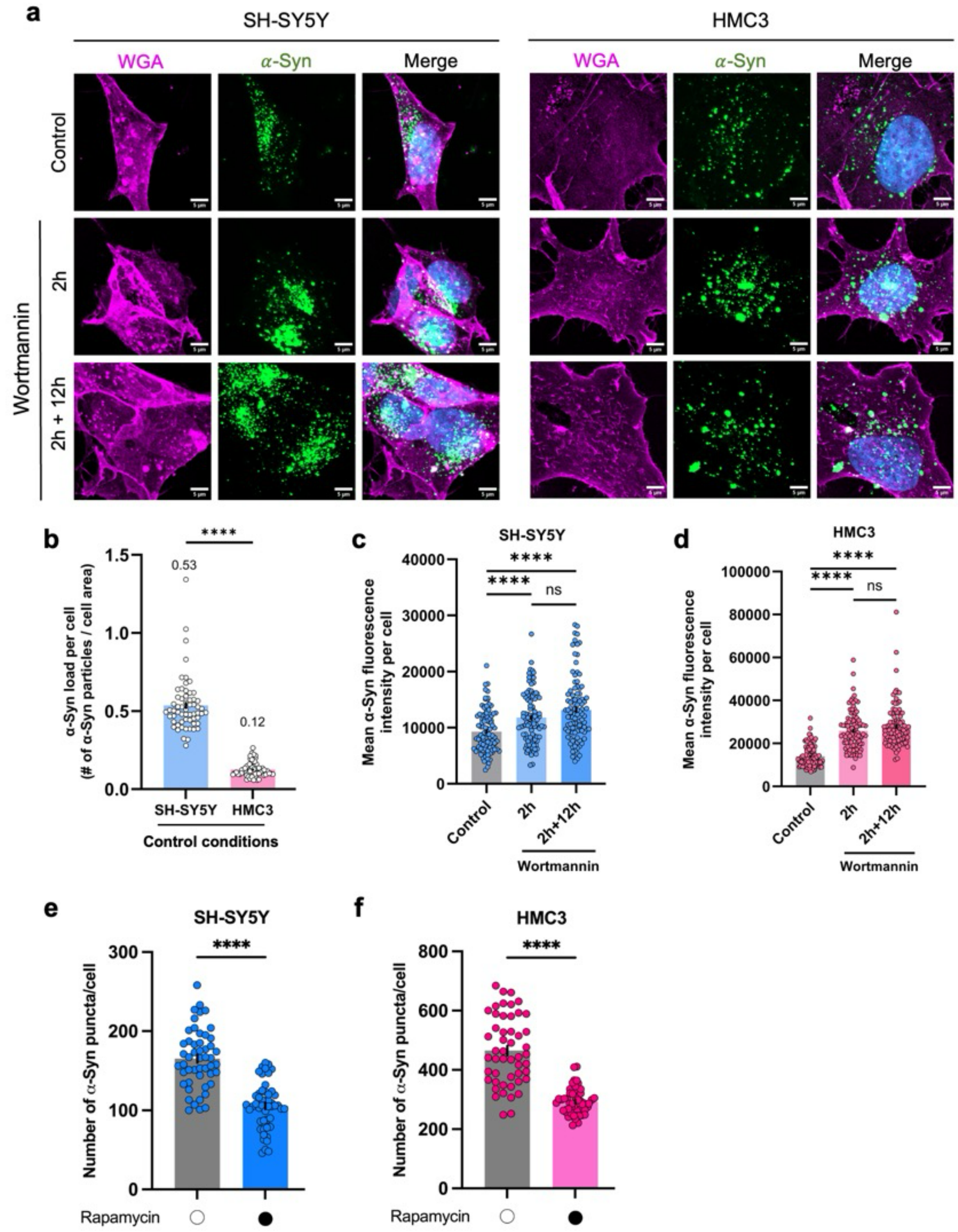

a
SH-SY5Y
HMC3
WGA
α-Syn
Merge
Control
Wortmannin
2h
2h + 12h
b
α-Syn load per cell (# of α-Syn particles / cell area)
0.53
0.12
SH-SY5Y
HMC3
Control conditions
c
SH-SY5Y
Mean α-Syn fluorescence intensity per cell
ns
Control
2h
2h+12h
Wortmannin
d
HMC3
Mean α-Syn fluorescence intensity per cell
ns
Control
2h
2h+12h
Wortmannin
e
SH-SY5Y
Number of α-Syn puncta/cell
Rapamycin
f
HMC3
Number of α-Syn puncta/cell
Rapamycin

Supplementary Figure 5. Load of aggregates in neuronal cells and microglia upon wortmannin treatment. (**a**) Representative images of SH-SY5Y cells (left panels) and HMC3 cells (right panels) after 16h of exposure to α-Syn aggregates (control, upper panels), followed by 2 h treatment with wortmannin (2 h, middle panels), and grown for additional 12 h after washing wortmannin off (2 h + 12 h, lower panels). (**b**) Quantification of the absolute load of α-Syn aggregates in neuronal cells and microglia in control (no wortmannin treatment) conditions (number of α-Syn particles per unit area of cells). Mean values are mentioned within the graph. N=3, n=60 cells per group. Error bars represent SEM. Statistical significance was analyzed using two-sided Mann-Whitney test. ****$p<1x10^{-15}$. (**c** and **d**) Quantification of intracellular aggregate load in different conditions for SH-SY5Y (**c**) and HMC3 (**d**) cells. Data represented as absolute mean intensity values. N=3, n=100 cells per condition. Error bars represent SEM. Statistical significance was analyzed using Brown-Forsythe and Welch One-Way ANOVA with Games-Howell's multiple comparison. For (**c**): ns (p=0.1548); control versus 2h, ****$p=9.89x10^{-5}$; control versus 2+12h, ****$p=1.64X10^{-7}$. For (**d**): ns (p=0.2513); control versus 2h, ****$p<1x10^{-15}$; control versus 2+12h, ****$p=3.8x10^{-14}$. (**e** and **f**) Quantification of the number of α-Syn aggregates per neuronal (**e**) or microglial (**f**) cell in control conditions or upon treatment with rapamycin. N=3, n=50 cells per condition. Error bars represent SEM. Statistical significance was analyzed using two-sided Student's unpaired t-test with Welch's correction. For (**e**): ****$p=3.5x10^{-14}$; for (**f**): ****$p=2.5x10^{-14}$.

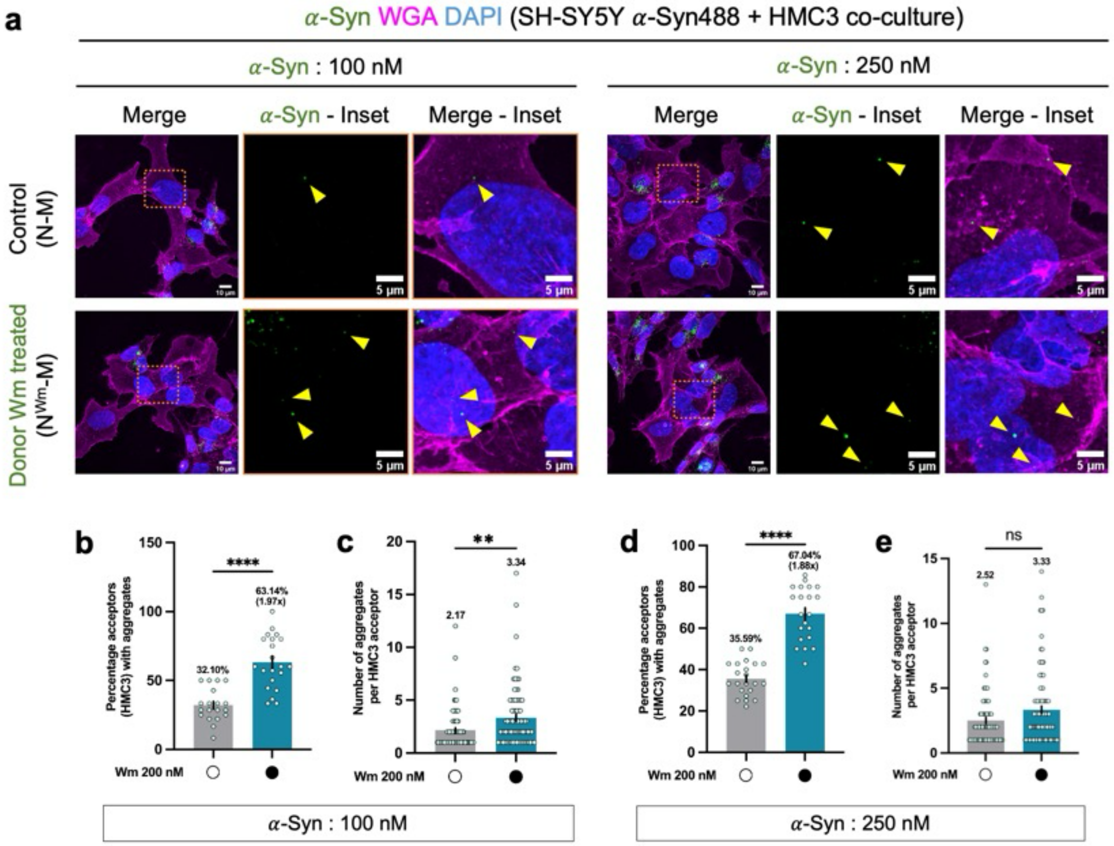

Supplementary Figure 6. Effect of α-Syn exposure concentrations on transfer from neuronal cells to microglia. (**a**) Representative images of 12h co-cultures between α-Syn loaded SH-SY5Y neuronal cells at different aggregate concentrations (100 nM and 250 nM) and HMC3 microglia. (**b**) Percentage of HMC3 acceptor cells that received aggregates for 100 nM dose of neuronal α-Syn exposure. N=3 independent experiments, n=149 acceptor cells for N-M, and 133 acceptor cells for N^{Wm}-M. Error bars represent SEM. Statistical significance was analyzed using unpaired two-sided Student's t-test with Welch's correction. ****p=4.69x10^{-7}. (**c**) Quantification of the number of aggregates received per cell by acceptor HMC3 in (**b**). N=3 independent experiments, n=47 acceptor cells for N-M, and 80 acceptor cells for N^{Wm}-M. Error bars represent SEM. Statistical significance was analyzed using two-sided unpaired Student's t-test with Welch's correction. ***p=0.0036. (**d**) Percentage of HMC3 acceptor cells that received aggregates

for 250 nM dose of neuronal α-Syn exposure. Average percentage of acceptor cells positive for aggregates is mentioned within the graph. N=3 independent experiments, n=173 acceptor cells for N-M, and 141 acceptor cells for N^{Wm}-M. Error bars represent SEM. Statistical significance was analyzed using two-sided unpaired Student's t-test with Welch's correction. ****$p=6.36x10^{-11}$. (**e**) Quantification of the number of aggregates received per cell by acceptor HMC3 in (**d**). Mean number of aggregates is mentioned within the graph. N=3 independent experiments, n=61 acceptor cells for N-M, and 92 acceptor cells for N^{Wm}-M. Error bars represent SEM. Statistical significance was analyzed using two-sided unpaired Student's t-test with Welch's correction. ns (p=0.1220).

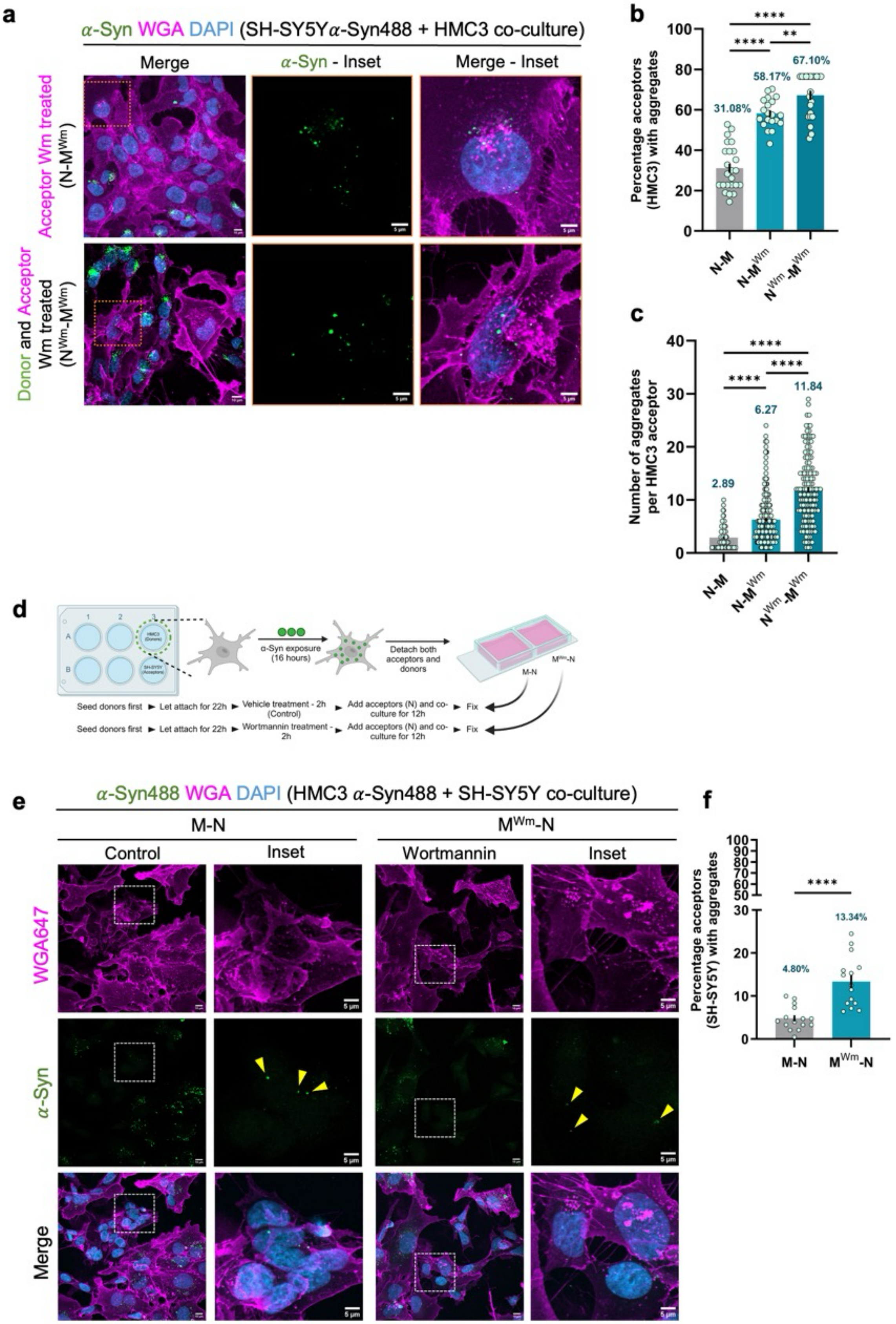
a
α-Syn WGA DAPI (SH-SY5Yα-Syn488 + HMC3 co-culture)
Merge
α-Syn - Inset
Merge - Inset
Acceptor Wm treated (N-M^Wm)
Donor and Acceptor Wm treated (N^Wm-M^Wm)
b
Percentage acceptors (HMC3) with aggregates
31.08%
58.17%
67.10%
N-M
N-M^Wm
N^Wm-M^Wm
c
Number of aggregates per HMC3 acceptor
2.89
6.27
11.84
N-M
N-M^Wm
N^Wm-M^Wm
d
HMC3 (Donors)
SH-SY5Y (Acceptors)
α-Syn exposure (16 hours)
Detach both acceptors and donors
M-N
M^Wm-N
Seed donors first ► Let attach for 22h ► Vehicle treatment - 2h (Control) ► Add acceptors (N) and co-culture for 12h ► Fix
Seed donors first ► Let attach for 22h ► Wortmannin treatment - 2h ► Add acceptors (N) and co-culture for 12h ► Fix
e
α-Syn488 WGA DAPI (HMC3 α-Syn488 + SH-SY5Y co-culture)
M-N
M^Wm-N
Control
Inset
Wortmannin
Inset
WGA647
α-Syn
Merge
f
Percentage acceptors (SH-SY5Y) with aggregates
4.80%
13.34%
M-N
M^Wm-N

Supplementary Figure 7. Contribution of autophagy in transfer of aggregates. (**a**) Representative images of 12h co-cultures between α-Syn loaded SH-SY5Y neuronal cells and HMC3 microglia (wortmannin treated in different combinations. Wortmannin treatments of respective cell populations are represented by the superscript “Wm”. Dotted box indicates the region of acceptor microglia zoomed in. (**b**) Percentage of HMC3 acceptor cells that received aggregates in control and wortmannin-treated co-cultures. Average percentage of acceptor cells positive for aggregates is mentioned within the graph. N=3 independent experiments, n=240 acceptor cells for N-M (same as in Figure 6b, c), 322 acceptor cells for N-M^{Wm}, and 161 acceptor cells for N^{Wm}-M^{Wm}. Error bars represent SEM. Statistical significance was analyzed using Brown-Forsythe and Welch One-Way ANOVA with Dunnett’s T3 multiple comparison. **$p=0.0092$, ****$p<1\times10^{-15}$. (**c**) Quantification of the number of aggregates received per cell by acceptor HMC3 in (**b**). Mean number of aggregates is mentioned within the graph. N=3 independent experiments, n=137 acceptor cells for N-M (same as in Figure 6b, d), 264 acceptor cells for N-M^{Wm}, and 144 acceptor cells for N^{Wm}-M^{Wm}. Error bars represent SEM. Statistical significance was analyzed using Kruskal-Wallis test with Dunn’s multiple comparison. ****$p<1\times10^{-15}$. (**d**) Schematic of co-culture experimental design to assess aggregate transfer from microglia to neuronal cells upon autophagy inhibition by Wm. Created in BioRender. Palese, F. (2026) https://BioRender.com/3zimbkm. (**e**) Representative images of co-culture between α-Syn loaded HMC3 cells (control – left panels and wortmannin treated – right panels) and SH-SY5Y neuronal cells. Dotted box indicates the region zoomed in. Yellow arrowheads point towards the aggregates. (**f**) Percentage of SH-SY5Y acceptor cells that received aggregates in control (M-N) and wortmannin-treated HMC3 (M^{Wm}-N) co-cultures. Average percentage of acceptor cells positive for aggregates mentioned in red within the graph. N=3 independent experiments, n=322 acceptor cells for control and 327 acceptor cells for wortmannin treated groups. Error bars represent SEM. Statistical significance was analyzed using two-sided unpaired Student’s t-test with Welch’s correction. ****$p=5.87\times10^{-5}$.

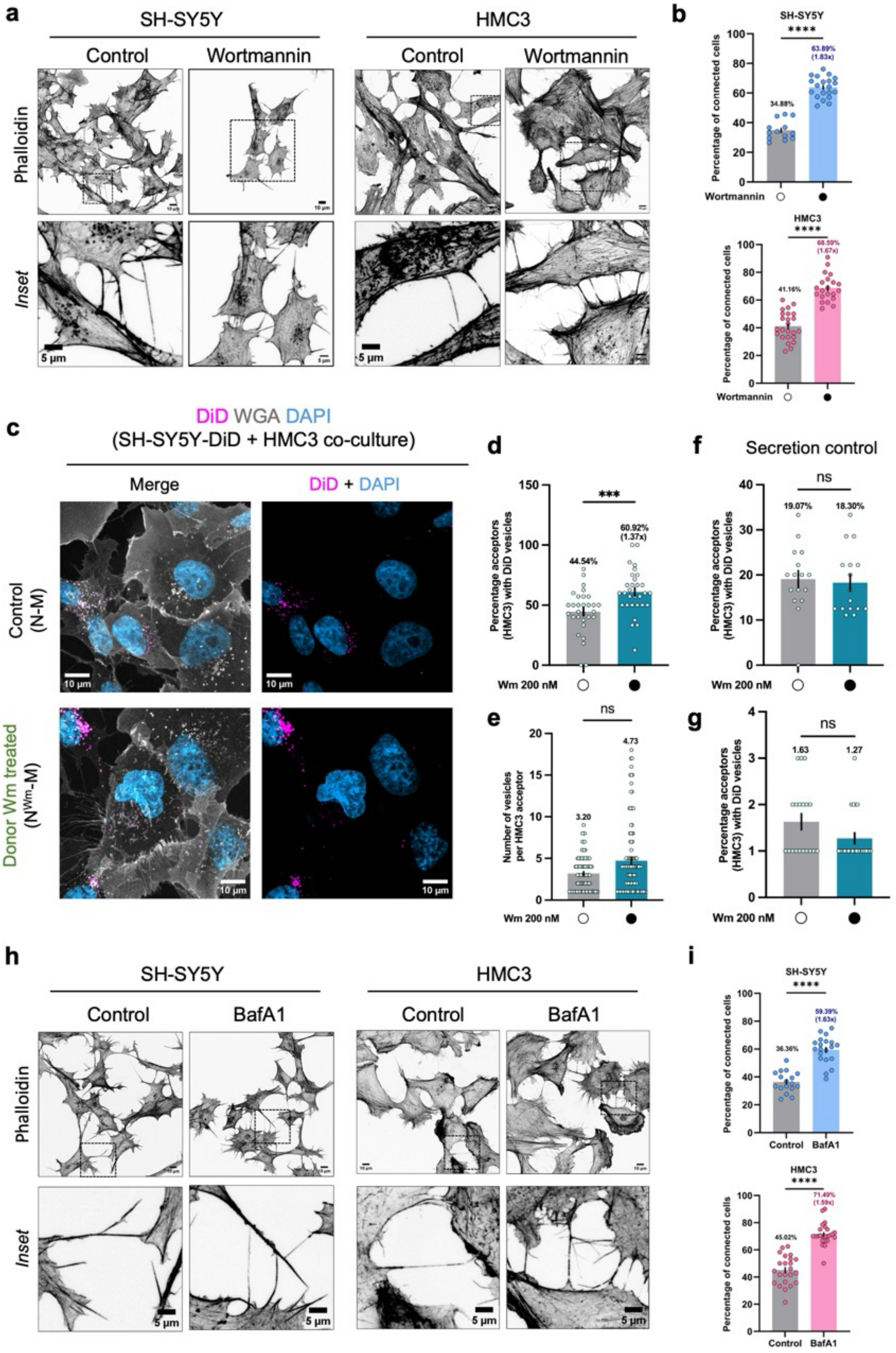

a
SH-SY5Y
HMC3
Control
Wortmannin
Phalloidin
Inset
5 µm
b
SH-SY5Y
****
Percentage of connected cells
34.88%
63.89% (1.83x)
Wortmannin
HMC3
41.16%
68.59% (1.67x)
c
DiD WGA DAPI
(SH-SY5Y-DiD + HMC3 co-culture)
Merge
DiD + DAPI
Control (N-M)
Donor Wm treated (N^{Wm}-M)
10 µm
d
***
Percentage acceptors (HMC3) with DiD vesicles
44.54%
60.92% (1.37x)
Wm 200 nM
e
ns
Number of vesicles per HMC3 acceptor
3.20
4.73
f
Secretion control
ns
19.07%
18.30%
g
ns
1.63
1.27
h
SH-SY5Y
HMC3
Control
BafA1
Phalloidin
Inset
5 µm
i
SH-SY5Y
****
36.36%
59.39% (1.63x)
HMC3
45.02%
71.49% (1.59x)
Control
BafA1

Supplementary Figure 8: Autophagy inhibition and intercellular connections. (**a**) Representative images of control and wortmannin-treated SH-SY5Y and HMC3 cells, stained for phalloidin. (**b**) Percentage of connected SH-SY5Y and HMC3 cells. N=3; for SH-SY5Y: n=551 cells for control, and 641 cells for wortmannin treated group; for HMC3: n=368 cells for control, and 275 cells for wortmannin treated groups. Error bars represent SEM. Statistical significance was analyzed using two-sided unpaired Student's t-test with Welch's correction. ****p=2.23×10^{-12} for SH-SY5Y, and ****p=9.41×10^{-12} for HMC3. (**c**) Representative images of 12h co-cultures between DiD-dye-loaded SH-SY5Y and HMC3 microglia. (**d**) Percentage of DiD-positive HMC3 acceptors. N=3, n=204 for N-M, and 178 for N^{Wm}-M. Error bars represent SEM. Statistical significance was analyzed using two-sided Mann-Whitney test. ***p=0.0006. (**e**) Number of DiD+ vesicles received per acceptor HMC3 in (**d**). N=3, n=91 for N-M, and 106 for N^{Wm}-M. Error bars represent SEM. Statistical significance was analyzed using two-sided Mann-Whitney test. ns (p=0.0999). (**f**) Percentage of HMC3 acceptor cells that received DiD+ vesicles from neuronal cells via conditioned media. N=3, n=99 acceptor cells for N-M, and 123 acceptor cells for N^{Wm}-M. Error bars represent SEM. Statistical significance was analyzed using two-sided Mann-Whitney test. ns (p=0.4138). (**g**) Number of DiD+ vesicles received per acceptor HMC3 in (**f**). N=3, n=19 acceptor cells for N-M, and 22 acceptor cells for N^{Wm}-M. Error bars represent SEM. Statistical significance was analyzed using two-sided Mann-Whitney test. ns (p=0.0982). (**h**) Representative images of SH-SY5Y and HMC3 cells treated or not with bafilomycin A1 and stained with phalloidin. (**i**) Percentage of connected SH-SY5Y and HMC3 cells. N=3; for SH-SY5Y: n=398 cells for control and 500 cells for bafilomycin A1 treated condition; for HMC3: n=205 cells for control and 215 cells for bafilomycin A1 treated condition. Statistical significance was analyzed using two-sided unpaired Student's t-test with Welch's correction. ****p=1.59×10^{-11} for SH-SY5Y, and ****p=5.95×10^{-13} for HMC3.

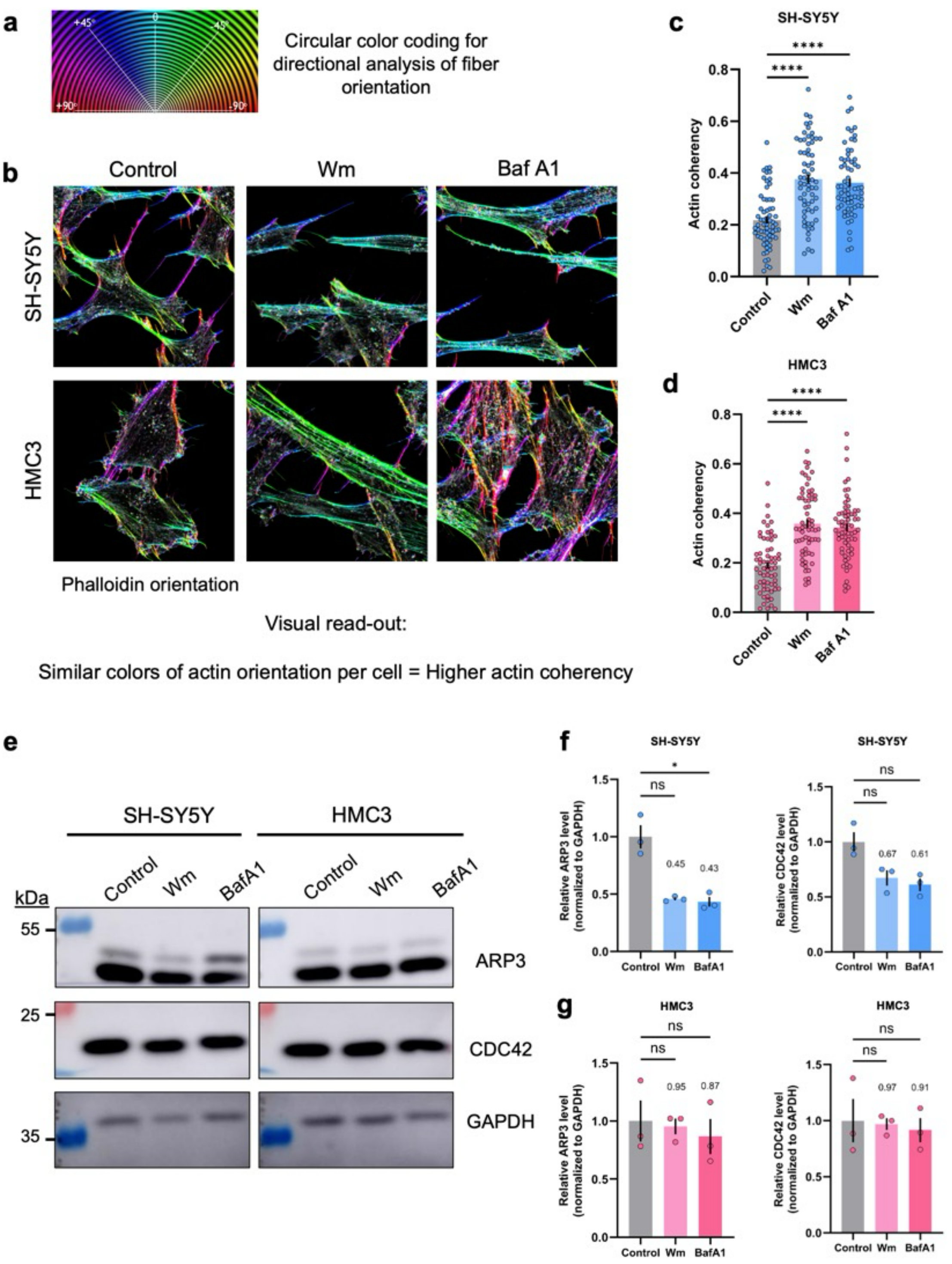

Supplementary Figure 9. Actin re-organization upon autophagy compromise. (**a**) Color code for orientation analysis of actin fibers – colors represent the direction of orientation,

ranging from +90° to -90°. Image courtesy of Urszula Zajaczkowska, obtained from EPFL biomedical imaging group webpage for OrientationJ (https://bigwww.epfl.ch/demo/orientation/). (**b**) Representative images of orientation of actin fibers in neuronal cells (upper panels) and microglia (lower panels). Similar colors of actin directionalities in each cell represents a higher degree of coherency. (**c** and **d**) Quantification of coherency of actin fiber alignment in neuronal cells (**c**) and microglia (**d**) upon Wm and Baf A1 treatments. N=3 independent experiments, n=65 cells per group. Error bars represent SEM. Statistical significance was analyzed using Kruskal-Wallis test with Dunn's multiple comparison. For (**c**): control versus Wm, ****$p=6.40 \times 10^{-10}$; control versus Baf A1, $p=6.01 \times 10^{-9}$. For (**d**): control versus Wm, $p=5.83 \times 10^{-11}$; control versus Baf A1, $p=7.85 \times 10^{-10}$. (**e**) Representative immunoblots of ARP3 and CDC42 in neuronal cells and microglia upon treatment with the autophagy inhibitor wortmannin (Wm) and flux blocker Baf A1. (**f**) Quantification of the relative ARP3 and CDC42 levels in neuronal cells normalized to GAPDH. N=3 independent experiments. Error bars represent SEM. Statistical significance was analyzed using Brown-Forsythe and Welch One-Way ANOVA with Dunnett's T3 multiple comparisons. For ARP3 analysis: control versus Wm: ns – p=0.0689; control versus Baf A1: *p=0.0323. For CDC42 analysis: control versus Wm: ns – p=0.1033; control versus Baf A1: ns – p=0.0787. (**g**) Quantification of the relative ARP3 and CDC42 levels in microglia normalized to GAPDH. N=3 independent experiments. Error bars represent SEM. Statistical significance was analyzed using Brown-Forsythe and Welch One-Way ANOVA with Dunnett's T3 multiple comparisons. For ARP3 analysis: control versus Wm: ns – p=0.9914; control versus Baf A1: ns – p=0.9159. For CDC42 analysis: control versus Wm: ns – p=0.9977; control versus Baf A1: ns – p=0.9708.

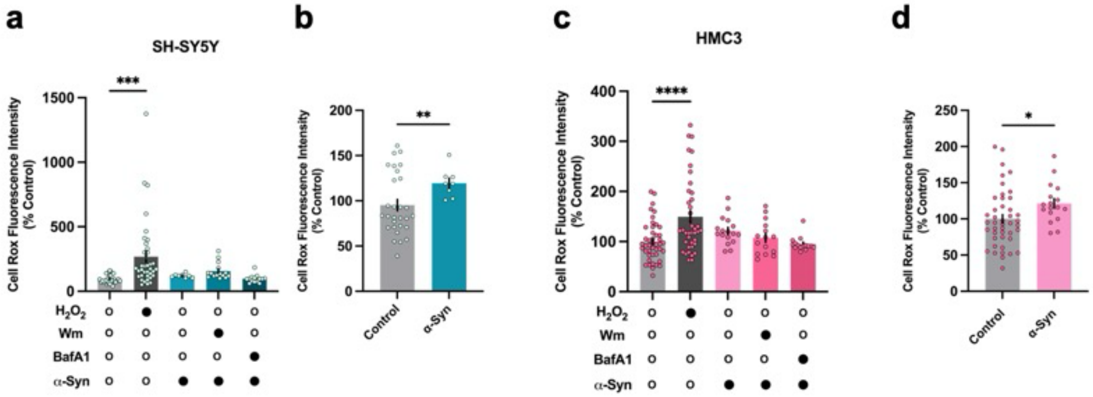

Supplementary Figure 10. Reactive Oxygen Species accumulation upon α-Syn and autophagic blockade. (**a and c**) Quantification of intracellular ROS levels in neuronal cells and microglia under control conditions, following H_2O_2 treatment (250 μM, 1h), or after exposure to α-Syn (500 nM, 16h) alone or in combination with wortmannin (200 nM, 2h) or bafilomycin A1 (400 nM, 4h). N=3 independent experiments. Error bars represent SEM. Statistical significance was analyzed using One-way ANOVA followed by Dunnett's multiple comparison test. For (**a**), ***p=0.0002, and for (**c**), ****p=5.43x10^{-5}. (**b and d**) Quantification of ROS levels in control conditions or following α-Syn treatment. Statistical significance was assessed using two-sided unpaired Student's t-test with Welch's correction. For (**b**), **p=0.0086, and (**d**) *p=0.0211.
