# Supplementary material for: Impaired α-Synuclein aggregate clearance in neuronal cells drive their spread to microglia through tunneling nanotubes
Source: Nat Commun. 2026 Mar 12;17:3832. doi: 10.1038/s41467-026-69930-y (PMC13121836; doi:10.1038/s41467-026-69930-y)
Supplement: Supplementary file 2 — Description of Additional Supplementary File [file 41467_2026_69930_MOESM2_ESM.pdf]

Description of Additional Supplementary Files

Supplementary Movie 1. Tracking lysosomal movement in control condition of SH-SY5Y cells. Lysosomes were labeled with Lysotracker Green for 30 minutes at 37 °C before acquiring time-lapse images. Lysosomes are depicted in gray-invert LUT.

Supplementary Movie 2. Tracking lysosomal movement upon $\boldsymbol{\alpha}$-Syn treatment of SH-SY5Y cells. Representative time-lapse movie of neuronal lysosomes associated with α-Syn aggregate (red). Lysosomes were labeled with Lysotracker Green for 30 minutes at 37 °C before acquiring time-lapse images. Lysosomes are depicted in gray-invert LUT.

Supplementary Movie 3. Tracking lysosomal movement upon $\boldsymbol{\alpha}$-Syn treatment of SH-SY5Y cells. Representative time-lapse movie of neuronal lysosomes not associated with α-Syn aggregate (red) in an α-Syn-exposure condition. Lysosomes were labeled with Lysotracker Green for 30 minutes at 37 °C before acquiring time-lapse images. Lysosomes are depicted in gray-invert LUT.

Supplementary Movie 4. Tracking lysosomal movement in control condition of HMC3 cells. Lysosomes were labeled with Lysotracker Green for 30 minutes at 37 °C before acquiring time-lapse images. Lysosomes are depicted in gray-invert LUT.

Supplementary Movie 5. Tracking lysosomal movement upon $\boldsymbol{\alpha}$-Syn treatment of HMC3 cells. Representative time-lapse movie of neuronal lysosomes associated with α-Syn aggregate (red). Lysosomes were labeled with Lysotracker Green for 30 minutes at 37 °C before acquiring time-lapse images. Lysosomes are depicted in gray-invert LUT.

Supplementary Movie 6. Tracking lysosomal movement upon $\boldsymbol{\alpha}$-Syn treatment of HMC3 cells. Representative time-lapse movie of neuronal lysosomes not associated with α-Syn aggregate (red) in an α-Syn-exposure condition. Lysosomes were labeled with Lysotracker Green for 30 minutes at 37 °C before acquiring time-lapse images. Lysosomes are depicted in gray-invert LUT.
