# Supplementary material for: Impaired α-Synuclein aggregate clearance in neuronal cells drive their spread to microglia through tunneling nanotubes
Source: Nat Commun. 2026 Mar 12;17:3832. doi: 10.1038/s41467-026-69930-y (PMC13121836; doi:10.1038/s41467-026-69930-y)
Supplement: Supplementary file 9 — Reporting summary [file 41467_2026_69930_MOESM9_ESM.pdf]

nature portfolio

Corresponding author(s): Chiara Zurzolo
Last updated by author(s): Jan 21, 2026

Reporting Summary

Nature Portfolio wishes to improve the reproducibility of the work that we publish. This form provides structure for consistency and transparency in reporting. For further information on Nature Portfolio policies, see our Editorial Policies and the Editorial Policy Checklist.

Statistics

For all statistical analyses, confirm that the following items are present in the figure legend, table legend, main text, or Methods section.

n/a	Confirmed	
☐	☒	The exact sample size (*n*) for each experimental group/condition, given as a discrete number and unit of measurement
☐	☒	A statement on whether measurements were taken from distinct samples or whether the same sample was measured repeatedly
☐	☒	The statistical test(s) used AND whether they are one- or two-sided<br>*Only common tests should be described solely by name; describe more complex techniques in the Methods section.*
☒	☐	A description of all covariates tested
☐	☒	A description of any assumptions or corrections, such as tests of normality and adjustment for multiple comparisons
☐	☒	A full description of the statistical parameters including central tendency (e.g. means) or other basic estimates (e.g. regression coefficient) AND variation (e.g. standard deviation) or associated estimates of uncertainty (e.g. confidence intervals)
☐	☒	For null hypothesis testing, the test statistic (e.g. *F*, *t*, *r*) with confidence intervals, effect sizes, degrees of freedom and *P* value noted<br>*Give P values as exact values whenever suitable.*
☒	☐	For Bayesian analysis, information on the choice of priors and Markov chain Monte Carlo settings
☒	☐	For hierarchical and complex designs, identification of the appropriate level for tests and full reporting of outcomes
☒	☐	Estimates of effect sizes (e.g. Cohen's *d*, Pearson's *r*), indicating how they were calculated

Our web collection on statistics for biologists contains articles on many of the points above.

Software and code

Policy information about availability of computer code

Data collection	Fluorescent Images were acquired using Zen Blue (LSM confocal), Zen Black (Elyra 7 SM) softwares of Zeiss, or Nikon NIS-Elements software of Nikon Eclipse Ti2 spinning disk confocal microscope.
Data analysis	Image analyses was performed using FIJI. Statistical analyses were performed and graphs were prepared in GraphPad Prism 10.0.

For manuscripts utilizing custom algorithms or software that are central to the research but not yet described in published literature, software must be made available to editors and reviewers. We strongly encourage code deposition in a community repository (e.g. GitHub). See the Nature Portfolio guidelines for submitting code & software for further information.

Data

Policy information about availability of data

All manuscripts must include a data availability statement. This statement should provide the following information, where applicable:

- Accession codes, unique identifiers, or web links for publicly available datasets
- A description of any restrictions on data availability
- For clinical datasets or third party data, please ensure that the statement adheres to our policy

All data are presented as main figures, or supplementary figures.

Research involving human participants, their data, or biological material

Policy information about studies with human participants or human data. See also policy information about sex, gender (identity/presentation), and sexual orientation and race, ethnicity and racism.

Reporting on sex and gender	NA
Reporting on race, ethnicity, or other socially relevant groupings	NA
Population characteristics	NA
Recruitment	NA
Ethics oversight	NA

Note that full information on the approval of the study protocol must also be provided in the manuscript.

Field-specific reporting

Please select the one below that is the best fit for your research. If you are not sure, read the appropriate sections before making your selection.

☒ Life sciences ☐ Behavioural & social sciences ☐ Ecological, evolutionary & environmental sciences

For a reference copy of the document with all sections, see nature.com/documents/nr-reporting-summary-flat.pdf

Life sciences study design

All studies must disclose on these points even when the disclosure is negative.

Sample size	No statistical method was used to predetermine sample size. Sample size was determined empirically and was based on previous experience from similar studies. In vitro experiments were performed including at least 3 biological replicates to confirm reproducibility. Sample or group sizes of the experiments were chosen based on previous experience and published studies.
Data exclusions	No data points/biological replicates were excluded from analyses.
Replication	All experiments were repeated atleast 3 times, with independent biological replicates.
Randomization	Treatment groups were designated randomly between wells and plates.
Blinding	Investigators were not blinded during experiments and analyses. There were defined groups and blinding was not necessary

Reporting for specific materials, systems and methods

We require information from authors about some types of materials, experimental systems and methods used in many studies. Here, indicate whether each material, system or method listed is relevant to your study. If you are not sure if a list item applies to your research, read the appropriate section before selecting a response.

Materials & experimental systems

n/a	Involved in the study	
☐	☒	Antibodies
☐	☒	Eukaryotic cell lines
☒	☐	Palaeontology and archaeology
☒	☐	Animals and other organisms
☒	☐	Clinical data
☒	☐	Dual use research of concern
☒	☐	Plants

Methods

n/a	Involved in the study	
☒	☐	ChIP-seq
☒	☐	Flow cytometry
☒	☐	MRI-based neuroimaging

Antibodies

Antibodies used	mouse anti-LAMP1 (Developmental Studies Hybridoma Bank, H4A3; 1:100), rabbit anti-LC3 (Medical and Biological Laboratories International Corporation, PM036; 1:400), guinea pig anti-p62 (Progen, GP62-C; 1:400), and rabbit anti-TFEB (Cell Signaling

	Technology, 4240; 1:100), mouse anti-human Galectin3 (Clone 194804, MAB1154; 1:50), rabbit anti-human IST1 (Proteintech, 19842-1-AP; 1:100), anti-LC3B, D11 XP #3868, Cell Signaling Technology, 1:1000; anti-phospho S211-TFEB, E9S8N #37681, Cell Signaling Technology, 1:1000; anti-phospho S235/236-S6 ribosomal protein, D57.2.2E XP #4858, Cell Signaling Technology, 1:1000; anti-S6 ribosomal protein, 5G10 #2217, Cell Signaling Technology, 1:1000; anti CDC42, ab64533, abcam, 1:1000; anti-ARP3, A5979, Sigma-Aldrich, 1:2000; anti-β-TUBULIN, PA1-41331, Thermo Fisher Scientific, 1:5000; and anti-GAPDH antibody Sigma, G9545, 1:5000
Validation	All antibodies used were commercially available, validated, and cited by various studies. Webpage links for the listed antibodies with confirmed species reactivity are mentioned below:<br>mouse anti-LAMP1 (Developmental Studies Hybridoma Bank, H4A3) - Confirmed Species Reactivity: Hamster, Human, Primate, Rat (https://dshb.biology.uiowa.edu/H4A3)<br>rabbit anti-LC3 (Medical and Biological Laboratories International Corporation, PM036) - Species Reactivity: Human, Mouse, Rat, Hamster, Zebrafish (https://www.mblbio.com/bio/g/dtl/A/?pcd=PM036)<br>guinea pig anti-p62 (Progen, GP62-C) - Reactivity: Bovine, Human, Mouse, Rat (https://www.progen.com/anti-p62-SQSTM1-C-terminus-guinea-pig-polyclonal-serum/GP62-C)<br>rabbit anti-TFEB (Cell Signaling Technology, 4240) - Species Reactivity: Human (https://www.cellsignal.com/products/primary-antibodies/tfeb-antibody/4240?srsltid=AfmBOoqAd5Plq3AXkJ68RmX6EoCJ_YmD3kklRe68NreIyOrebLpMbgxL)<br>mouse anti-human Galectin3 (Clone 194804, MAB1154) - Species reactivity: Human (https://www.rndsystems.com/products/human-galectin-3-antibody-194804_mab1154)<br>rabbit anti-human IST1 (Proteintech, 19842-1-AP) - Tested reactivity: human, mouse, rat (https://www.ptglab.com/products/OLC1-Antibody-19842-1-AP.htm?srsltid=AfmBOooxF0XNi9Fu4emD0ulC2a-YfgdAlEyDiHLfIsXmtieRPgaX1sth)<br>anti-LC3B, D11 XP #3868, Cell Signaling Technology - Specoes reactivity: Human (https://www.cellsignal.com/products/primary-antibodies/lc3b-d11-xp-rabbit-mab/3868?srsltid=AfmBOopBs2fd0v_Wo4yYRXxUHUsn9RpATriMRyyGgs0oSapBqDaU6GUE)<br>anti-phospho S211-TFEB, E9S8N #37681 - Species reactivity: Human (https://www.cellsignal.com/products/primary-antibodies/phospho-tfeb-ser211-e9s8n-rabbit-mab/37681?srsltid=AfmBOooccBKO_n0ZzODrqRYnt_h6xj_p1HNWy1vXlSOItX2YOAewBvx)<br>anti-phospho S235/236-S6 ribosomal protein, D57.2.2E XP #4858, Cell Signaling Technology - Species reactivity: Human, Mouse, Rat, Monkey, Mink, S. cerevisiae (https://www.cellsignal.com/products/primary-antibodies/phospho-s6-ribosomal-protein-ser235-236-d57-2-2e-xp-rabbit-mab/4858?srsltid=AfmBOoruq22krIKXEFOTi0LJKeOkXUuOvFAzBCUrsE3yaOS5viIKeRUF)<br>anti-S6 ribosomal protein, 5G10 #2217, Cell Signaling Technology - Species reactivity: Human, Mouse, Rat, Monkey (https://www.cellsignal.com/products/primary-antibodies/s6-ribosomal-protein-5g10-rabbit-mab/2217?srsltid=AfmBOoq-VcJxVaaqTl9bAVsgPlH-8ApAoS2Be4lR09IhUjpfxizrMIv-)<br>anti CDC42, ab64533, abcam - Species reactivity: Mouse, Human (https://www.abcam.com/en-us/products/primary-antibodies/cdc42-antibody-ab64533?srsltid=AfmBOoq2mnrd8yHs67CKWruYKq0mVZHHrjIJinDTX1mqhqBesENGVfxK)<br>anti-ARP3, A5979, Sigma-Aldrich - Species reactivity: human, mouse, rat, canine (https://www.sigmaaldrich.com/FR/en/product/sigma/a5979?srsltid=AfmBOoosuyX4QYTi6919jIOIIufR-Kb_OwEjLqM6fll45QuWrd02Vjf7)<br>anti-β-TUBULIN, PA1-41331, Thermo Fisher Scientific - Species reactivity: Bovine, Dog, Chicken, Cat, Human, Mouse, Non-human primate, Rat, Xenopus (https://www.thermofisher.com/antibody/product/beta-Tubulin-Antibody-Polyclonal/PA1-41331)<br>anti-GAPDH antibody Sigma, G9545 - Species reactivity: mouse, rat, human (https://www.sigmaaldrich.com/FR/en/product/sigma/g9545?srsltid=AfmBOoplfrdIoD75P9ldlCbBn6Je0N_nCEDx4n7_sHWhNClcySXs6M-u)

Eukaryotic cell lines

Policy information about cell lines and Sex and Gender in Research

Cell line source(s)	All cell lines were sourced from ATCC. Human iPSC lines used in our studies were generated following procedures approved by the Commission on Guarantees concerning the Donation and Use of Human Tissues and Cells of the Carlos III Health Institute, Madrid, Spain.
Authentication	All the cells lines used in this study were authenticated by the supplier. iPSC-derived neurons and microglia were validated by immunofluorescence experiments.
Mycoplasma contamination	Cells were not tested for mycoplasma contamination regularly. However, periodic testings of cell lines used in the lab, including those in this study, were conducted.
Commonly misidentified lines<br>(See ICLAC register)	No misidentified cell lines were used in the study.

Plants

Seed stocks	NA
Novel plant genotypes	NA
Authentication	NA
